# A simple AI-enabled method for quantifying bacterial adhesion on dental materials

**DOI:** 10.1080/26415275.2022.2114479

**Published:** 2022-08-31

**Authors:** Hao Ding, Yunzhen Yang, Xin Li, Gary Shun-Pan Cheung, Jukka Pekka Matinlinna, Michael Burrow, James Kit-Hon Tsoi

**Affiliations:** a Dental Materials Science, Applied Oral Sciences and Community Dental Care, Faculty of Dentistry, The University of Hong Kong, Pokfulam, Hong Kong; b Restorative Dental Sciences, Faculty of Dentistry, The University of Hong Kong, Pokfulam, Hong Kong; c Department of Stomatology Shenzhen University General Hospital, Shenzhen University Clinical Medical Academy, Shenzhen, China

**Keywords:** Bacteria, artificial intelligence, zirconia, PMMA, dental materials

## Abstract

**Purpose: **Measurement of bacterial adhesion has been of great interest for different dental materials. Various methods have been used for bacterial counting; however, they are all indirect measurements with estimated results and therefore cannot truly reflect the adhesion status. This study provides a new direct measurement approach by using a simple artificial intelligence (AI) method to quantify the initial bacterial adhesion on different dental materials using Scanning Electron Microscope (SEM) images. **Materials and Methods:** *Porphyromonas gingivalis* (*P.g.*) and *Fusobacterium nucleatum* (*F.n.*) were used for bacterial adhesion on dental zirconia surfaces, and the adhesion was evaluated using SEM images at time points of one, seven, and 24 h(s). Image pre-processing and bacterial area measurement were performed using Fiji software with a machine learning plugin. The same AI method was also applied on SEM with *Streptococcus mutans* (*S.m.*) inoculated PMMA nano-structured surface at 1, 24, 72, and 168 h(s), and then further compared with the CLSM method. **Results: **For both *P.g.* and *F.n.* initiation adhesion on zirconia, a new linear correlation (r^2^ > 0.98) was found between bacteria adhered area and time, such that: $\sqrt{b\mathrm{acteria}\;\mathrm{adhered}\;\mathrm{area}\;({mm}^{2})}\propto \log\left(\mathrm{time}\right)$

For S.m. on PMMA surface, live/dead staining CLSM method and the newly proposed AI method on SEM images were strongly and positively associated (Pearson's correlation coefficient *r* > 0.9), i.e. both methods are comparable. **Conclusions:** SEM images can be analyzed directly for both morphology and quantifying bacterial adhesion on different dental materials’ surfaces by the simple AI-enabled method with reduced time, cost, and labours.

## 1. Introduction

Oral biofilm plays an important role in the pathogenesis of oral diseases such as dental caries and periodontal diseases. Oral diseases do not only affects oral health, but also general health and quality of life that consequently creating a financial burden due to the high treatment costs and that may not covered by universal health coverage [1]. Bacterial adhesion is the primary step in biofilm formation, which is a complicated process that is affected by numerous factors such as bacterial properties, material surface characteristics, and environment [2]. Biofilm can be formed on any surface within an oral cavity (i.e. dental plaque), causing dental caries, periodontal diseases, and failure of dental materials due to, say, peri-implantitis [3–5].

Research on bacterial growth has been studied for decades. Conceptually, this is a simple process because most bacterial growth follows binary fission. Typically, bacterial growth on material *in vitro* follow a growth curve that includes four phases: (1) the (initial) lag phase: bacteria is maturing and metabolically active before the start of exponential growth; (2) the exponential (or log) phase: bacteria is growing at a constant rate; (3) the stationary phase: the growth rate of the bacteria is equal to the death rate due to limited nutrients; (4) and the death phase: a decrease in live bacteria due to lack of nutrients [6]. However, unlike controlled laboratory conditions, intraoral conditions such as environment, nutrients, temperature, and moisture levels are dynamic and diversified. Thus, the bacterial growth phases may coexist and overlap within the same biofilm. As such, the bacterial or biofilm growth and activity may be described more realistically as adhesion, growth, maturation and dispersion. This superimposition of phases makes it challenging when investigating the behavior of bacterial growth on materials surfaces. In fact, studying initial bacterial adhesion on the tooth or dental material surfaces [7–9] is of vital importance, because this can better understand the various types of bacterial adherence on different surfaces for anti-bacterial strategies of dental materials. Thus, the mechanisms of bacteria-material interaction and how bacteria react on different surfaces can be explored.

To evaluate the antibacterial properties of a material, many bacteria counting methods have been published, including (A) direct counting methods, such as plate counts, transmitted light microscopy, standard fluorescence microscopy, and flow cytometry; (B) indirect counting methods such as colony-forming unit (CFU) plate count, radiolabeling, spectrophotometry, ATP marker, and nucleic acid probes [10]. It is worth noting that each method has its own advantages for specific purposes. Take CFU as an example; it is the most widely used indirect counting method for evaluating antibacterial properties. It is also simple and does not require high-end equipment. However, there are some disadvantages. For example, only the culturable fraction of a biofilm population can be detected by CFU, the method is limited to detecting microorganisms that develop colonies on agar plates, and it is a time- and labor-consuming process [11].

Alternatively, direct counting methods have been introduced to solve the problems encountered by indirect methods. Scanned electron microscopy (SEM) provides excellent quality images of bacteria and biofilms with high resolution and magnification. However, it cannot quantify the number of bacteria cells and biofilms, and as such, has limited its use in this field. Confocal laser scanning microscopy (CLSM) is another widely used direct counting method – both morphologically and quantitatively. It enables the analysis of biological structures, without damaging the biological structure [12]. Nevertheless, it is fluorescence based, and thus fluorescent dyes are required before observing. Therefore, the natural fluorescence may interfere with the testing results. Additionally, the high cost associated with CLSM may limit its use.

In recent years, artificial intelligence (AI) has gained a great amount of global interest. It was first coined in 1956 by John McCarthy as ‘the science and engineering of making intelligent machines, especially intelligent computer programs’ [13]. The term and concept have contributed a lot to this new research area since then. After the two ‘AI Winters’ in the late 1970s and early 1990s, AI research had reached a bottleneck, which led to many ups and downs and a lack of funding support. From the late 1990s, AI research gradually regained focus from researchers, together with the development of computational power, many fields have been benefited from AI since then [14]. John Searle [15] further classified AI as being ‘strong’ or ‘weak’ (narrow) depending on whether a machine could achieve consciousness. Current AI applications are all narrow-based, which can be attributed to solving specific problems and tasks. Notably, the application of AI and big data in industry has presented the fourth industrial revolution. There are different ways in which a goal can be achieved utilizing AI, the most important among them are machine learning (ML), natural language processing, image processing, computer vision, speech, and robotics.

In image processing, segmentation is the process of partitioning a digital image into two or more segments [16]. Using AI and ML algorithms, automatic segmentation of radiology imaging, including X-ray, computed tomography (CT), magnetic resonance imaging (MRI), positron emission tomography (PET), and ultrasound imaging become possible, and results have shown the AI/ML-assisted automatic segmentation would enable a more accurate and efficient diagnosis of tumors and cancers in human organs [17,18]. Recently, AI techniques have been utilized in dentistry in various ways [19] such as predicting the debonding probability of CAD/CAM composite resin crowns [20] and assisting in orthodontic treatment planning [21].

In a bacterial counting assessment, Andreini et al. [22] and Ferrari et al. [23] utilized convolutional neural networks (CNNs), which is an ML approach, to automate bacterial counting on agar plates. Nonetheless, most AI approaches require certain knowledge of programming. Hence, it is not feasible for researchers with little or no programming knowledge to develop such approaches. Here, we explore the usage of an easy, free and assessable AI in the initial bacterial counting assessment by direct quantification of bacteria on SEM images using an ImageJ-based plug-in. This is a new method which provides a direct perceived result of the initial bacterial counting assessment, and more information can be acquired from the results, *i.e.* the bacterial-surface interaction.

## 2. Materials and methods

In order to test and verify the broad applicability of the new image analysing method using AI, two different sets of SEM data were used that include (A): *Porphyromonas gingivalis* (*P.g.*) and *Fusobacterium nucleatum* (*F.n.*) inoculated on a flat zirconia surface, and (B): *Streptococcus mutans* (*S.m.*) inoculated on an nano-structured Poly(methyl methacrylate) (PMMA) surface.

**Figure 1.**
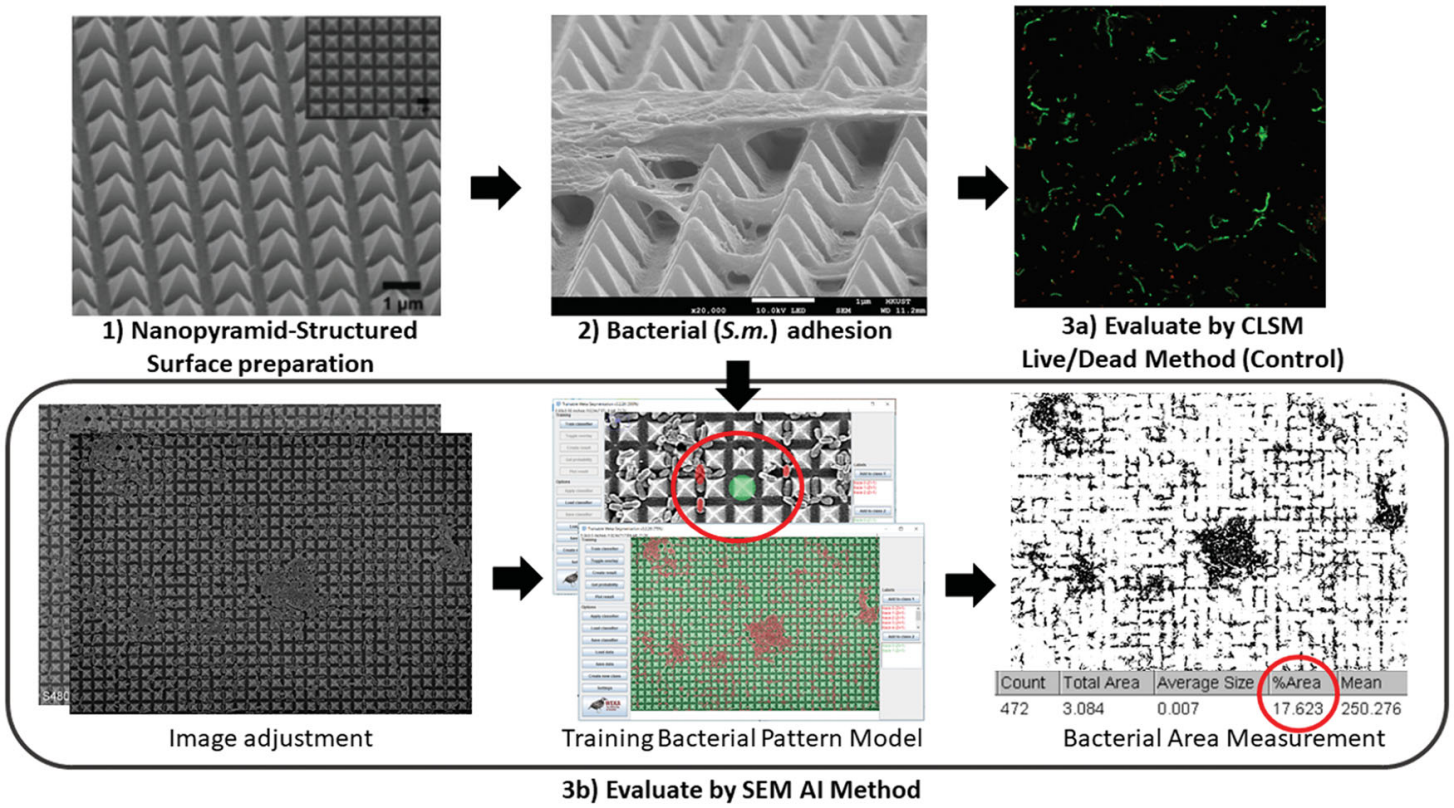
Schematics of (B): two initial bacterial adhesion counting methods.

For (A), it followed the protocols described in sections 2.1 to 2.3 before image processing, as stated in sections 2.4 to 2.5. For (B), the SEM and confocal microscopy (CSLM) data were adopted from previously published studies [24], whereas CLSM was used as control. In brief, the nano-structured PMMA surface was prepared *via* a molding process, and *S.m.* was used for the bacterial growth on the said surface (Figure 1). Bacterial growth was evaluated at respective time points of one hour, one day, three days, and seven days. The LIVE/DEAD BacLight™ Bacterial Viability Kit (L7012 Invitrogen; Molecular Probes, Eugene, OR, USA) was used for staining. Six areas were randomly selected and imaged by a CLSM (IX81 FluoView FV1000; Olympus, Shinjuku-ku, Tokyo, Japan). SYTO 9 was excited by a 488 nm laser and propidium iodide (PI) was excited by a 543 nm laser, a beam splitter SDM560 and a filter set BA655-755 was employed to observe the viability and distribution of biofilm on specimens. CLSM images were then imported to image analysis software (ImageJ; National Institutes of Health, Bethesda, Maryland, USA) and converted to 8-bit binary (black and white) images by adjusting the threshold. ‘Analyze Particles’ function was used to count the total amount of live and dead bacterial cells. Mean values of bacterial cell count in six areas were calculated for further comparison. The adopted SEM images in this study were processed using the protocol of sections 2.4 and 2.5.

### 2.1. Preparation of zirconia discs

Commercially available zirconia blocks (Ivoclar IPS e.max® ZirCAD MT A2, Lot:VM9002) of 98.5 mm in diameter and 14 mm in thickness were cut into discs of 25 mm in diameter and 1.2 mm in thickness by a high-speed linear precision saw (Isomet® 5000, BUEHLER, USA) with a diamond blade under running water. The samples were then polished with SiC abrasive papers of 1000-grit and 2000-grit in sequence on a polishing machine, and reached a surface roughness value of 0.5 µm. The polished zirconia blocks were sintered following the manufacturer’s protocol. The size of the sintered samples turned into 20.0 ± 0.5 mm in diameter and 1.0 mm in thickness due to the phase transformation during sintering. After the above treatments, the samples were ultrasonically cleaned with a 70% ethanol solution for 15 min, rinsed with double deionized water, and dried in clean ambient air for four hours before the samples were blown with clean high-pressure gas to remove any possible residue on the surface. The samples were finally sterilized with a dry heat method in 160 °C for two hours in a heating and drying oven (Model 600, MEMMERT, Germany).

A total of 24 cylindrical zirconia samples were randomly divided into six groups. A control group of four specimens was taken without any further treatment.

### 2.2. Bacteria inoculation

Two bacteria, *P.g.* (ATCC 33277) and *F.n.* (ATCC 23726), were selected for this study, both of which are common bacteria that appear in oral biofilm. To grow *P.g.* and *F.n.*, a medium of 10^8^ bacteria CFU/ml (*P.g.*/*F.n.*) in *P.g.* broth was added (1.5 ml/well) to 12-well plates (Corning, USA) with a zirconia disc in each well. The well plates were incubated anaerobically (incubation condition of CO_2_ of 10%, H_2_ of 10% and N_2_ of 80%) at 37 °C up to time points of one hour, seven hours, and 24 h. Grow medium (*P.g.* broth) composes of 30 g Tryptic Soy Broth (TSB), 5 g yeast extract, 1 L distilled water, and 10 ml hemin/vitamin K stock solution. TSB medium contains 17 g Tryptone, 3 g Phytone, 5 g NaCl, 2.5 g dipotassium phosphate (K_2_HPO_4_), 2.5 g glucose, and 1 L distilled water.

### 2.3. Scanning electron microscopy (SEM)

SEM (SU-1510, HITACHI, Japan) images were taken for the bacteria inoculated samples to observe the surface morphology, and then reserved for further image analysis. Images were taken at random locations at magnifications of 6000×, 4000×, 2000×, and 1000×.

Samples were rinsed with PBS solution to wash away any impurities from the broth, followed by fixation and dehydration procedures. Fixative solution of 2.5% glutaraldehyde was added to the well plates and kept in a refrigerator overnight. The ethanol solution with different concentrations (i.e. 70%, 85%, 95%, and 100%) were added to the well plates in sequence from low to high. The dehydrated time was 30 min for 70%, 85%, and 95% ethanol and 60 min for 100% ethanol. The samples were then dried for two hours and sputtered with 80% platinum and 20% palladium before SEM testing.

### 2.4. Image analysis

The SEM images of (A) and (B) were processed, and the initial bacterial growth was analyzed by the Trainable Weka Segmentation (TWS) plug-in in Fiji, an ImageJ based package, following the same procedure, as described below:

#### 2.4.1. Preprocessing

For all SEM images, the length and width are 2560 pixels and 1920 pixels. The lower part (2560 × 140 pixels) was trimmed because the scale bar could affect the results. Therefore, the size of the SEM images used for analysis has a dimension of 2560 × 1780. For (A), we choose the 2000 × magnification images for analysis because the morphology of the bacteria can be seen clearly under this magnification, yet a broader field of view is achieved. Thus, we can calculate that 810 pixels corresponds to 20 µm, according to the scale bar showed in the SEM images. For (B), 2500 × magnification was used, where 1012.5 pixels correspond to 20 µm.

To enhance the accuracy and efficiency of the process, the following image adjustment procedures were adopted: (1) Rolling ball background subtraction (to correct the unevenly illuminated background by using a ‘rolling ball’ algorithm); (2) Subtract background; (3) Contrast enhancement; (4) Remove outliers and (5) Image sharpening.

#### 2.4.2. Segmentation by TWS plug-in

**Figure 2.**
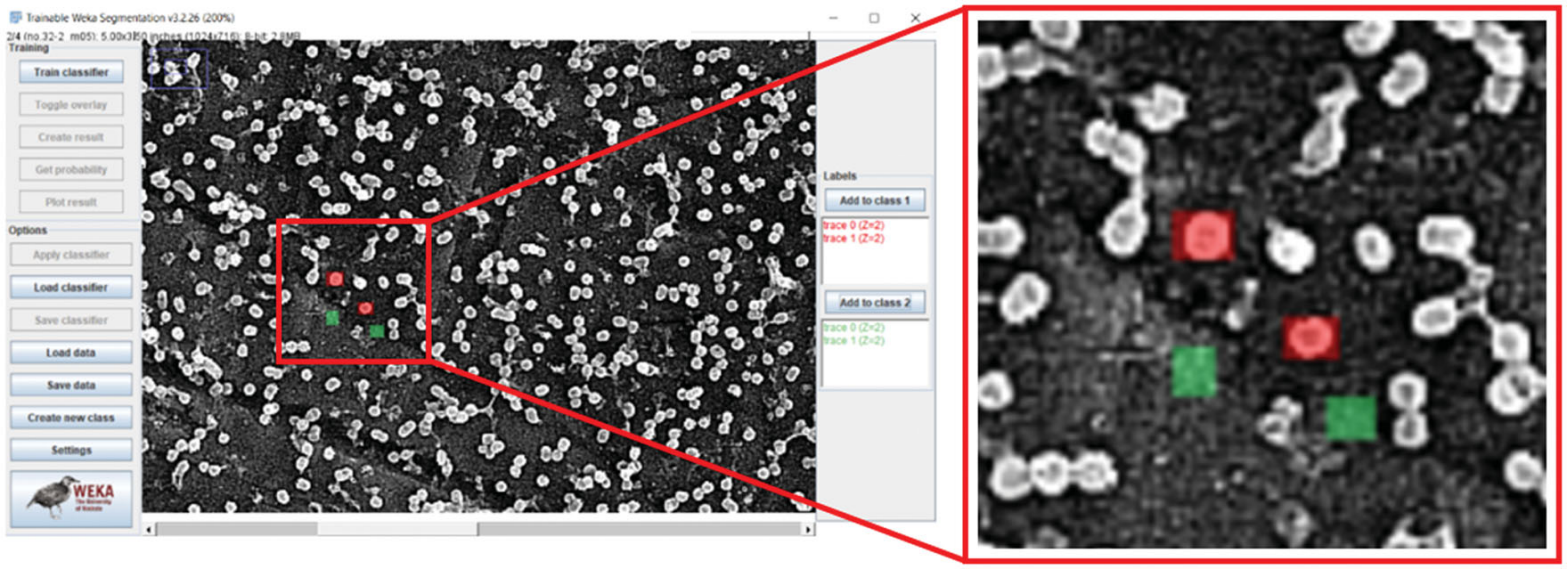
Demonstration of manually label the bacteria and background.

The adjusted images were then loaded into the TWS plug-in. Standard training settings were used. To train the ML algorithm (Figure 2), two classifiers were selected and used. Three to five areas of a bacteria occupied surface (classified as class 1 in red) and background (classified as class 2 in green) were manually marked, and then clicking ‘train classifier’, whereas the software would learn the pattern. Once finished, the generated result distribution would be overlaid on the image. Further adjustments regarding the accuracy of the computer segmented layers can be made if necessary.

The trained classifier can be saved by clicking ‘save classifier’. To reapply the classifier to subsequent images, click ‘load classifier’. It is noteworthy that ‘create new class’ enables the segmentation of three or more classes (*e.g.* a mixed bacteria culture).

#### 2.4.3. Results calculation

After image segmentation, the result was obtained by using the ‘create result’ function for further processing. An image in green and red would be created after clicking it. We further converted this image into an 8-bit black-and-white image; the black area on the image represents the bacteria occupied area. Then, the ‘analyze particles’ function can be used. The black area could be detected by this function, and the percentage of the black area occupied within the whole image could be calculated. In this study, particles below 50 pixels (for the SEM images of 2000× magnification) and 62.5 pixels (for the SEM images of 2500× magnification) were considered as noise, and therefore have been excluded from the results calculation.

An illustrative example for pre-processing, segmentation and calculation is shown in Supplementary Information.

### 2.5. Statistical analysis

For (B), the Pearson's correlation was used to measure the relationship between the results obtained by TWS Plug-in and CLSM methods for the initial bacterial adhesion stage (*i.e.* bacteria inoculated between 1 to 24 h) and the whole inoculation period (*i.e.* bacteria inoculated between 1 to 168 h), while the critical level for statistical significance was set at α = 0.05. The calculation was done using Excel software (Office 365, MICROSOFT, USA).

## 3. Results

### 3.1. P.g. And F.n. inoculated on the flat zirconia surface

**Figure 3.**
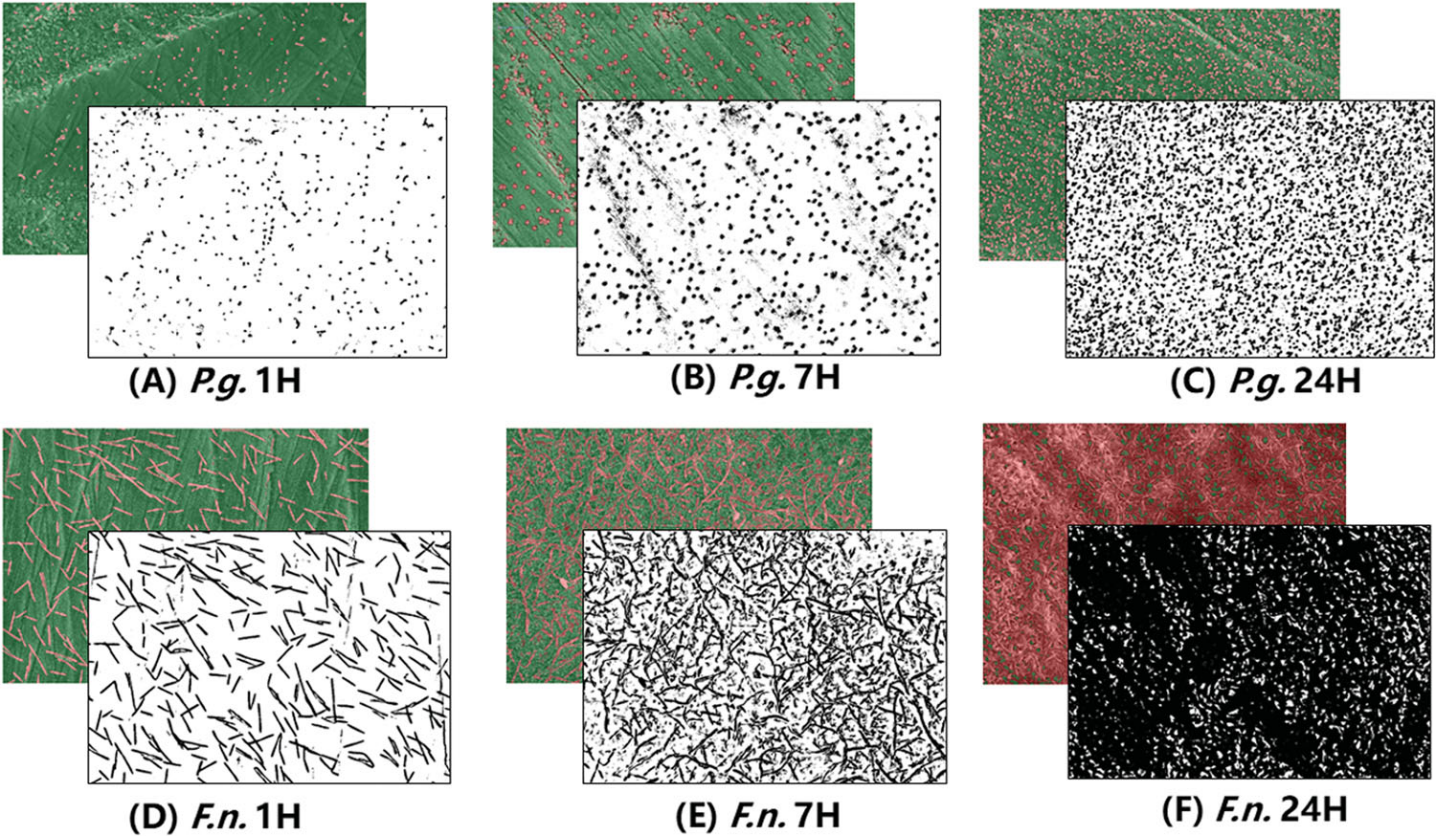
The area percentage of *P.g.* and *F.n.* occupied area after 1 h, 7 h and 24 h’ inoculation.

Figure 3 illustrates a representative example of overlapped SEM and 8-bit black-and-white images showing the distribution of *P.g.* and *F.n.* adhered on a flat zirconia surface at the time points of one hour, seven hours, and 24 h, respectively. The images were taken directly from the TWS plug-in, and a graduate growth for both *P.g.* and *F.n.* can be found over time. However, the growth rate varies. For instance, *P.g.* sample grows much slower than *F.n.* sample. For *F.n.* sample, the bacteria almost fully covered the zirconia surface after 24 h’ inoculation.

**Figure 4.**
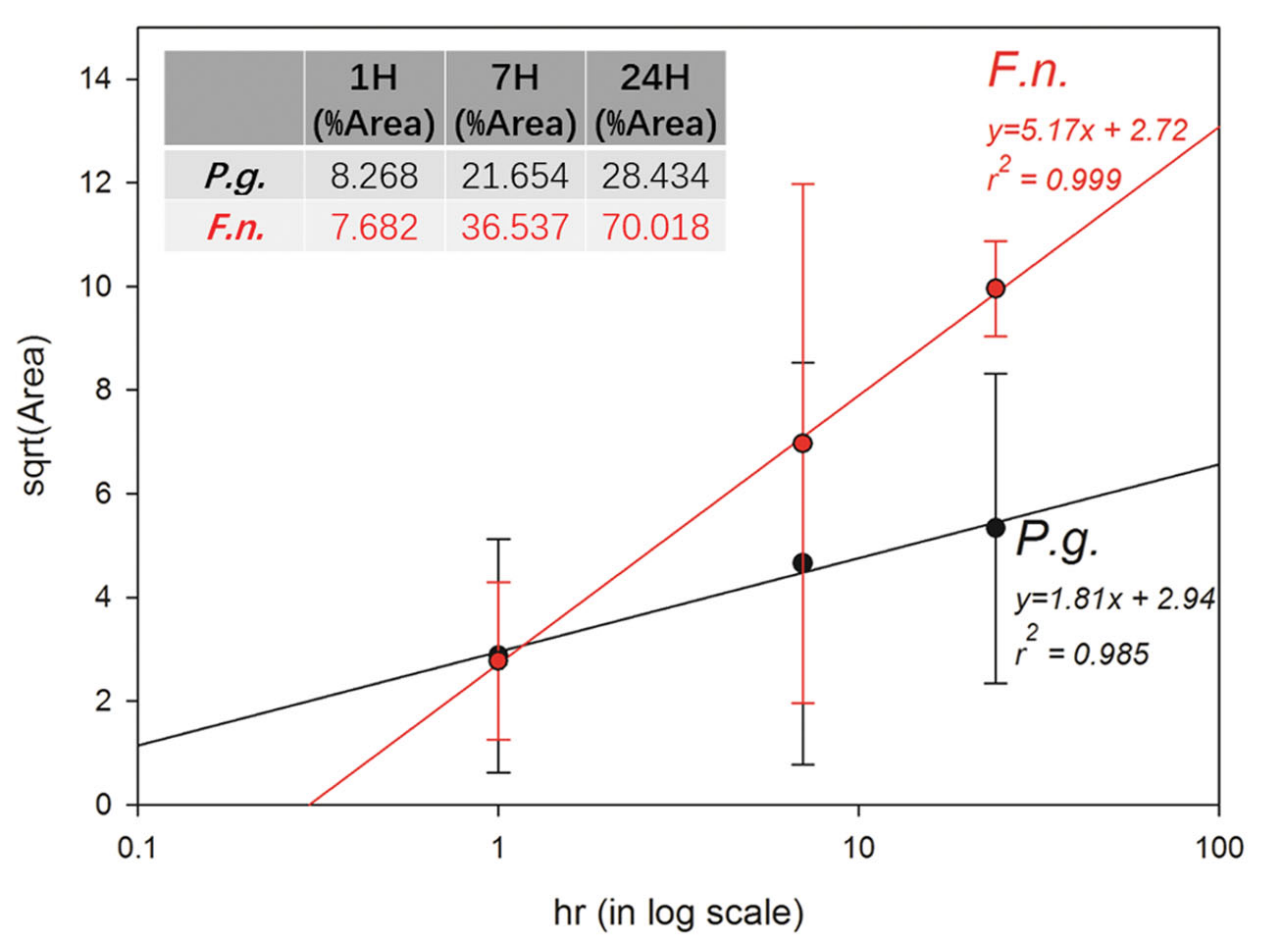
Area percentages of two bacteria in different time points and linear regression of the data.

Figure 4 shows the aera percentage of the *P.g.* and *F.n.* occupied area after one hour, seven hours, and 24 h’ inoculation. As Figure 4 shows, it can be concluded that strong linear correlations were found between the inoculation time (in log scale) and area percentage (in square root), that is,

$$\sqrt{b\mathrm{acteria}\;\mathrm{adhered}\;\mathrm{area}\;({mm}^{2})}\propto \log\left(\mathrm{time}\right)$$

The goodness of fit, R^2^ for this model were 0.985 and 0.999 for *P.g.* and *F.n.*, respectively.

### 3.2. S.m. Inoculated on the PMMA surface

In (B), the bacterial adhesion result generated by TWS plug-in were compared with the CLSM result at the same time points of one hour, 24 h, 72 h, and 168 h.

**Figure 5.**
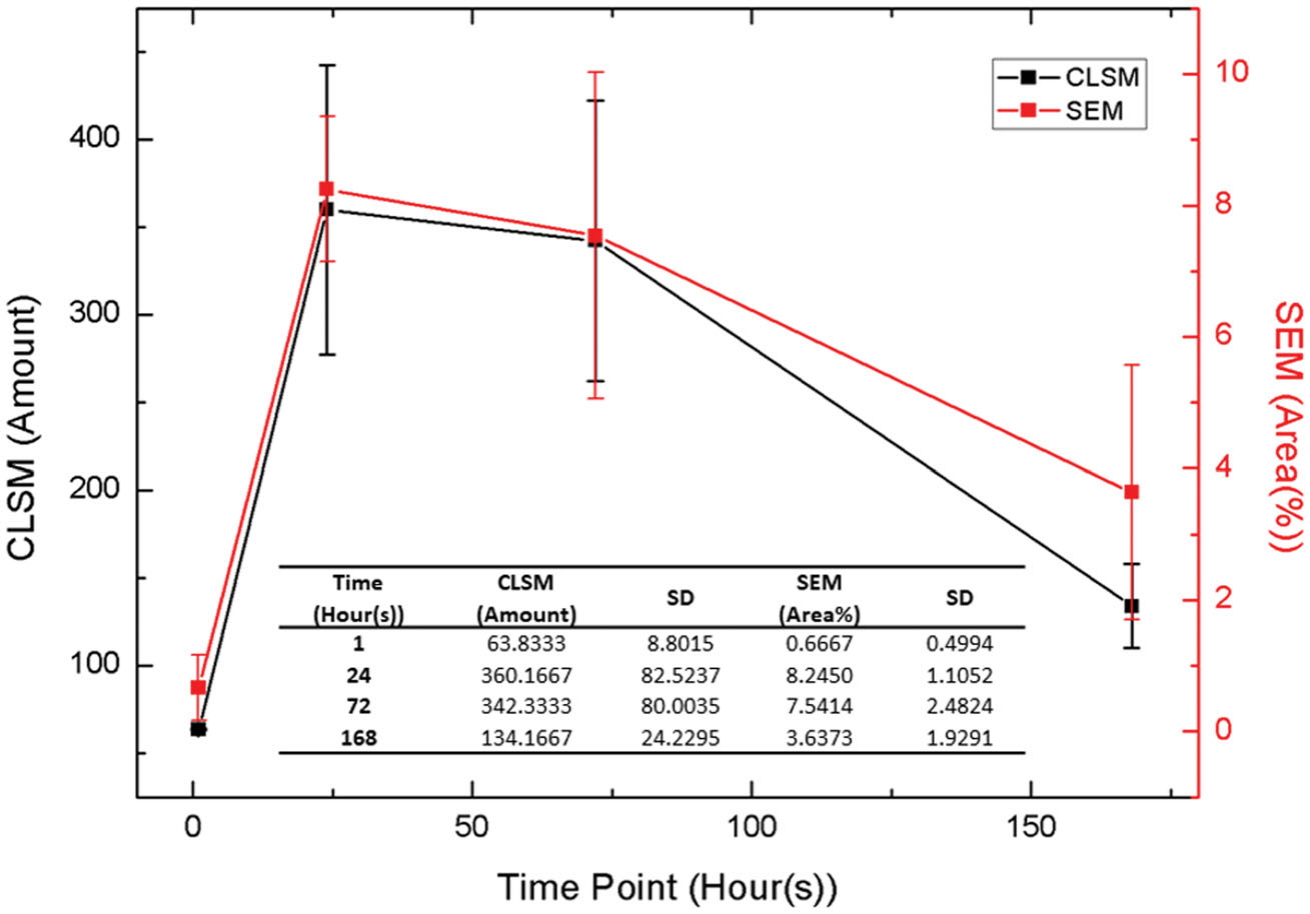
Summary of the bacterial adhesion results evaluated by CLSM (live plus died) and SEM images in different cell incubation times.

It can be seen from Figure 5 that comparable results were obtained after evaluating the bacterial adhesion between the live/dead staining method using CLSM images and the newly proposed AI method using SEM images. The Pearson's correlation coefficient (r) for the initial bacterial adhesion period (*S.m.* inoculated for 24 h) is calculated to be 0.926 and the p-value is 0.016; while the r for the whole inoculation period (0 to 168 h) is 0.903 with a p-value of 0.010, indicating both correlation coefficients are significantly different from zero. This said, the AI method can quantitatively analyse the SEM images and provide a good correlation that is comparable with CLSM.

## 4. Discussion

This article provides a new approach to evaluating initial bacterial adhesion on dental materials from 1 h to 7 days. In (A), we explored the use of TWS plug-in in the area of initial bacterial adhesion tests. The growth rate of *P.g.* sample is much slower than *F.n.* sample, this result is identical to other studies [25,26]. The main reason in the difference of growth rate may be due to the different nature of *P.g.* and *F.n.* with different specific growth rate (μ) and lag phase (λ) that are affected physically by various adhesion strength between different bacteria-material. In addition, it could also be affected by external influences such as increased deposition from the media. Further studies are needed in this aspect. In (B), we further compared the bacterial adhesion (*S.m.*) results generated by the TWS plug-in method and CLSM method, and a statistically significant correlation was found between the two methods. The TWS plug-in is an ease-of-use tool that enables researchers to perform images using an ML algorithm, even with limited programming experience.

Fiji is an open-source image processing package based on ImageJ. The TWS plug-in in Fiji was used for the image segmentation in this study. The TWS plug-in is a Fiji plugin and library that combines a collection of ML algorithms with a set of selected image features to produce pixel-based segmentations [27]. The TWS plug-in has been adopted in several kinds of studies such as tissue segmentation [28] and biofilm detection [29]. However, it has not been applied in a bacterial counting assessment.

There are some advantages to using this approach to examine initial bacterial adhesion. For instance, due to the high resolution of SEM images, it has become a practical method for observing the bacteria and morphology of testing samples in antimicrobial tests. The TWS plug-in utilizes SEM images as the source of bacterial counting. As such, this method could provide an option to make SEM images suitable for analyzing the bacterial adhesion not only qualitatively, but also (at least semi-) quantitatively that can complementary to traditional CFU counts and CLSM. Additionally, as shown in the results from this study, this AI-assisted method can apply on different dental materials with different surface profile (i.e. flat *vs.* nano-rough; zirconia *vs.* PMMA), and revealed new area-time relationship.

There are also certain limitations regarding this method. Since images obtained in SEM are two-dimensional (2 D), 2 D areas of surface-attached bacteria on materials can be detected by the TWS plug-in. Thus, only the initial stages of bacterial adhesion apply to this method. Once the testing surface is fully covered with bacteria, that is, 3 D biofilm is formed in whatever time, the program will not work since the biofilm has fully covered the testing surface. The thickness of the biofilm also cannot be detected by this method. In addition, this method does not differentiate between live and dead bacteria, as the analysis is based on SEM images. Thus, it may not be suitable for investigating live/dead related properties. Furthermore, according to the nature of the TWS plug-in (*i.e.* the pixel classification), a higher contrast of the image is helpful to differentiate the bacteria and background surface. In some cases, when the contrast of bacteria and background is low and difficult to identify, it may be difficult for the program to differentiate the two. To generate more accurate results, a higher contrast between the bacteria and background is recommended. Additionally, the bacteria may not grow homogeneously on testing surfaces, so it is necessary to capture more areas when taking SEM images to improve data accuracy.

The file size of SEM images uses a significant amount of computer memory, which can affect the efficiency or the time used in the ML training process for the TWS plug-in. In this study, the typical memory used for an SEM image is around 5 MB and takes around one minute for the program to finish the segmentation process. However, lower resolution images can greatly accelerate the speed of segmentation, but this also depends on the hardware settings of the computer itself. That said, human accuracy on training the images – particularly the marking of bacteria on surfaces using the TWS plugin – can also affect the applicability of the AI. In Fiji software, this includes rectangles, ovals, polygons, and freehand selections. To obtain the most accurate and uniform result, it is recommended to trace the bacteria using freehand selections with the margin closely attached to the bacteria periphery.

## 5. Conclusion

This study concluded that the AI tool (Fuji Trainable Weka Segmentation (TWS) plug-in) was able to measure the early stages of bacterial adhesion on dental materials with situations of (A) *Porphyromonas gingivalis* (*P.g.*) and *Fusobacterium nucleatum* (*F.n.*) on zirconia for 1, 7 and 24 h, and (B) *Streptococcus mutans* (*S.m.*) on nano-structured PMMA for 1, 24, 72, and 168 h(s), by direct quantifying the initial bacteria counts, i.e. occupied areas, from the respective SEM images. A new colonized area-time relationship about bacteria-zirconia was found, and the SEM images provided a closely match with CLSM results on PMMA.

## Supplementary Material

Supplemental Material

## References

[1] Flemmig TF, Beikler T. Control of oral biofilms. Periodontol 2000. 2011;55(1):9–15. 21134225 10.1111/j.1600-0757.2010.00383.x

[2] An YH, Friedman RJ. Concise review of mechanisms of bacterial adhesion to biomaterial surfaces. J. Biomed. Mater. Res. 1998;43(3):338–348. 9730073 10.1002/(sici)1097-4636(199823)43:3<338::aid-jbm16>3.0.co;2-b

[3] Sbordone L, Bortolaia C. Oral microbial biofilms and plaque-related diseases: microbial communities and their role in the shift from oral health to disease. Clin Oral Investig. 2003;7(4):181–188. 10.1007/s00784-003-0236-1 14598129

[4] Chen SY, Tsoi JKH, Tsang PCS, et al. Candida albicans aspects of binary titanium alloys for biomedical applications. Regen Biomater. 2020;7(2):213–220. 32296540 10.1093/rb/rbz052 PMC7147365

[5] Daood U, Banday N, Akram Z, et al. Mechanical and spectroscopic analysis of retrieved/failed dental implants. Coatings. 2017;7(11):201.

[6] Lund B, Baird-Parker AC, Baird-Parker TC, et al. Microbiological safety and quality of food. New York, NY: Springer Science & Business Media; 2000.

[7] Han AF, Tsoi JKH, Lung CYK, et al. An introduction of biological performance of zirconia with different surface characteristics: a review. Dent Mater J. 2020;39(4):523–530. 32507797 10.4012/dmj.2019-200

[8] Han AF, Li XL, Huang BX, et al. The effect of titanium implant surface modification on the dynamic process of initial microbial adhesion and biofilm formation. Int J Adhes Adhes. 2016;69:125–132.

[9] Tan CM, Tsoi JKH, Seneviratne CJ, et al. Evaluation of the Candida albicans removal and mechanical properties of denture acrylics cleaned by a low-cost powered toothbrush. J Prosthodont Res. 2014;58(4):243–251. 25052590 10.1016/j.jpor.2014.06.002

[10] Wilson C, Lukowicz R, Merchant S, et al. Quantitative and qualitative assessment methods for biofilm growth: a mini-review. Research and reviews. J Eng Technol. 2017;6(4). PMC6133255 30214915

[11] Azeredo J, Azevedo NF, Briandet R, et al. Critical review on biofilm methods. Crit Rev Microbiol. 2017;43(3):313–351. 27868469 10.1080/1040841X.2016.1208146

[12] Lawrence JR, Neu TR. Confocal laser scanning microscopy for analysis of microbial biofilms. Methods in enzymology: Elsevier, New York; 1999. p. 131–144. 10.1016/s0076-6879(99)10011-9 10547787

[13] Andresen SL. John McCarthy: father of AI. IEEE Intell. Syst. 2002;17(5):84–85.

[14] Silaparasetty N. An overview of artificial intelligence. Machine learning concepts with python and the jupyter notebook environment. 2020;3–19.

[15] Minds JS. Brains and programs. Behav Brain Sci. 1980;3:417–424.

[16] Zhang Z, Sejdić E. Radiological images and machine learning: Trends, perspectives, and prospects. Comput Biol Med. 2019;108:354–370. 31054502 10.1016/j.compbiomed.2019.02.017 PMC6531364

[17] Hsieh KL-C, Lo C-M, Hsiao C-J. Computer-aided grading of gliomas based on local and global MRI features. Comput Methods Programs Biomed. 2017;139:31–38. 28187893 10.1016/j.cmpb.2016.10.021

[18] Goyal M, Knackstedt T, Yan S, et al. Artificial intelligence-based image classification for diagnosis of skin cancer: Challenges and opportunities. Comput Biol Med. 2020;127:104065. 33246265 10.1016/j.compbiomed.2020.104065 PMC8290363

[19] Shan T, Tay F, Gu L. Application of artificial intelligence in dentistry. J Dent Res. 2021;100(3):232–244. 33118431 10.1177/0022034520969115

[20] Yamaguchi S, Lee C, Karaer O, et al. Predicting the debonding of CAD/CAM composite resin crowns with AI. J Dent Res. 2019;98(11):1234–1238. 31379234 10.1177/0022034519867641

[21] Li P, Kong D, Tang T, et al. Orthodontic treatment planning based on artificial neural networks. Sci Rep. 2019;9(1):1–9. 30765756 10.1038/s41598-018-38439-w PMC6375961

[22] Andreini P, Bonechi S, Bianchini M, et al. A deep learning approach to bacterial colony segmentation. International Conference on Artificial Neural Networks: Springer, Switzerland; 2018. p. 522–533.

[23] Ferrari A, Lombardi S, Signoroni A. Bacterial colony counting with convolutional neural networks in digital microbiology imaging. Pattern Recognit. 2017;61:629–640.

[24] Li X, Tsui K-H, Tsoi JK, et al. A nanostructured anti-biofilm surface widens the efficacy against spindle-shaped and chain-forming rod-like bacteria. Nanoscale. 2020;12(36):18864–18874. 32897280 10.1039/d0nr03809a

[25] Tavares LJ, Klein MI, Panariello BHD, et al. An in vitro model of Fusobacterium nucleatum and Porphyromonas gingivalis in single-and dual-species biofilms. J Periodontal Implant Sci. 2018;48(1):12–21. 29535887 10.5051/jpis.2018.48.1.12 PMC5841263

[26] Park JH, Lee J-K, Um H-S, et al. A periodontitis-associated multispecies model of an oral biofilm. J Periodontal Implant Sci. 2014;44(2):79–84. 24778902 10.5051/jpis.2014.44.2.79 PMC3999356

[27] Arganda-Carreras I, Kaynig V, Rueden C, et al. Trainable weka segmentation: a machine learning tool for microscopy pixel classification. Bioinformatics. 2017;33(15):2424–2426. 28369169 10.1093/bioinformatics/btx180

[28] Polan DF, Brady SL, Kaufman RA. Tissue segmentation of computed tomography images using a random Forest algorithm: a feasibility study. Phys Med Biol. 2016;61(17):6553–6569. 27530679 10.1088/0031-9155/61/17/6553 PMC5039942

[29] Vyas N, Sammons R, Addison O, et al. A quantitative method to measure biofilm removal efficiency from complex biomaterial surfaces using SEM and image analysis. Sci Rep. 2016;6:32694. 27601281 10.1038/srep32694 PMC5013386

