# Supplementary material for: A simple AI-enabled method for quantifying bacterial adhesion on dental materials
Source: Biomater Investig Dent. 2022 Aug 31;9(1):75–83. doi: 10.1080/26415275.2022.2114479 (PMC9448434; doi:10.1080/26415275.2022.2114479)
Supplement: Supplemental Material [file IABO_A_2114479_SM4407.docx]

**A Simple AI-enabled Method for Quantifying Bacterial Adhesion on Dental Materials**

**Supplementary Information** – **A Demonstration on Using TWS Plugin in Bacterial Counting Assessment**

All images were processed and analysed by Fiji (an open source image processing package based on ImageJ). The Trainable Weka Segmentation Plug-in in Fiji software was used for the image segmentation. The aim of segmentation is to count the number of bacteria and calculate the percentage of bacteria occupied area.

The raw images were adjusted as follows:

1. Stack the images if multiple images need to be analysed (Image 🡪 Stacks 🡪 Images to Stack);
2. Crop the image to remove the part with scale bar (Image 🡪 Crop);
3. Convert the image into 8-bit B&W format (Image 🡪 Type 🡪 8-bit);
4. Subtract background with following settings (Process 🡪 Subtract Background);


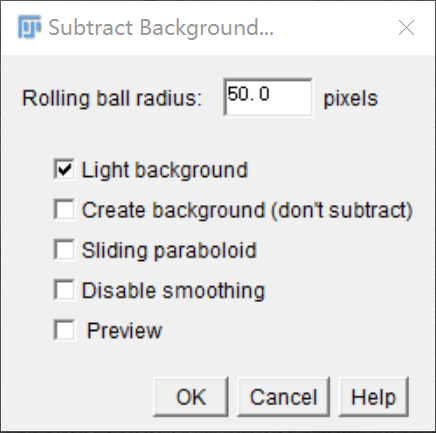


Figure S1 Settings of subtract background.

1. Remove outliers (Process 🡪 Noise 🡪 Remove outliers);


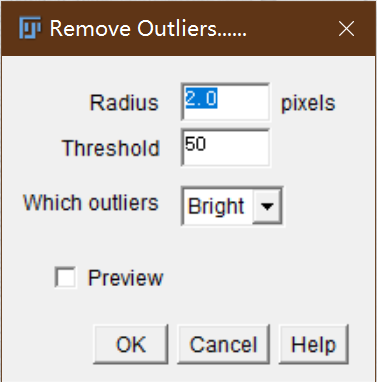


Figure S2 Settings of remove outliers.

1. Enhance contrast (Process 🡪 Enhance contrast).


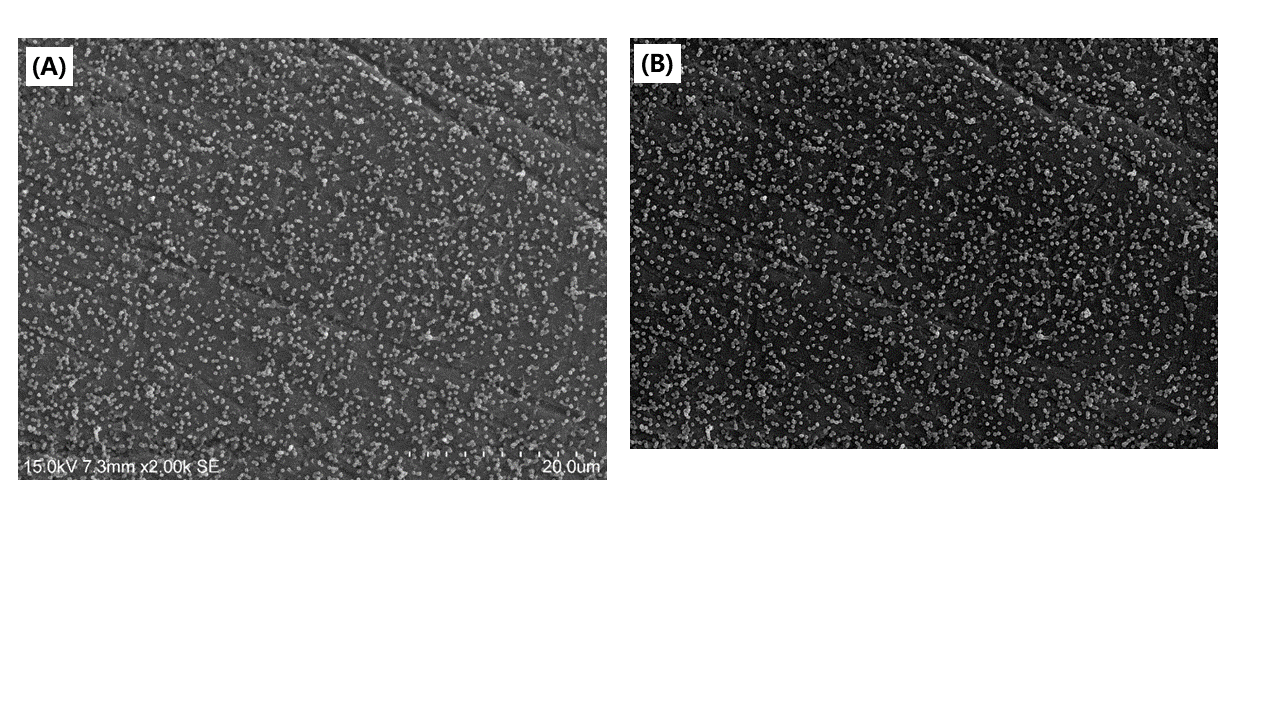


Figure S3 SEM images (A) before and (B) after the adjustments*.*

Then the image would be ready to be segmented. Trainable Weka Segmentation (Plugin 🡪 Segmentation 🡪 Trainable Weka Segmentation) was used for this purpose. To train the machine learning algorithm, manually mark one to two areas of bacteria occupied areas (classified as class 1 in red) and background (classified as class 2 in green), and then click “Train classifier”, the computer would learn the pattern (as Figure S4 shows).


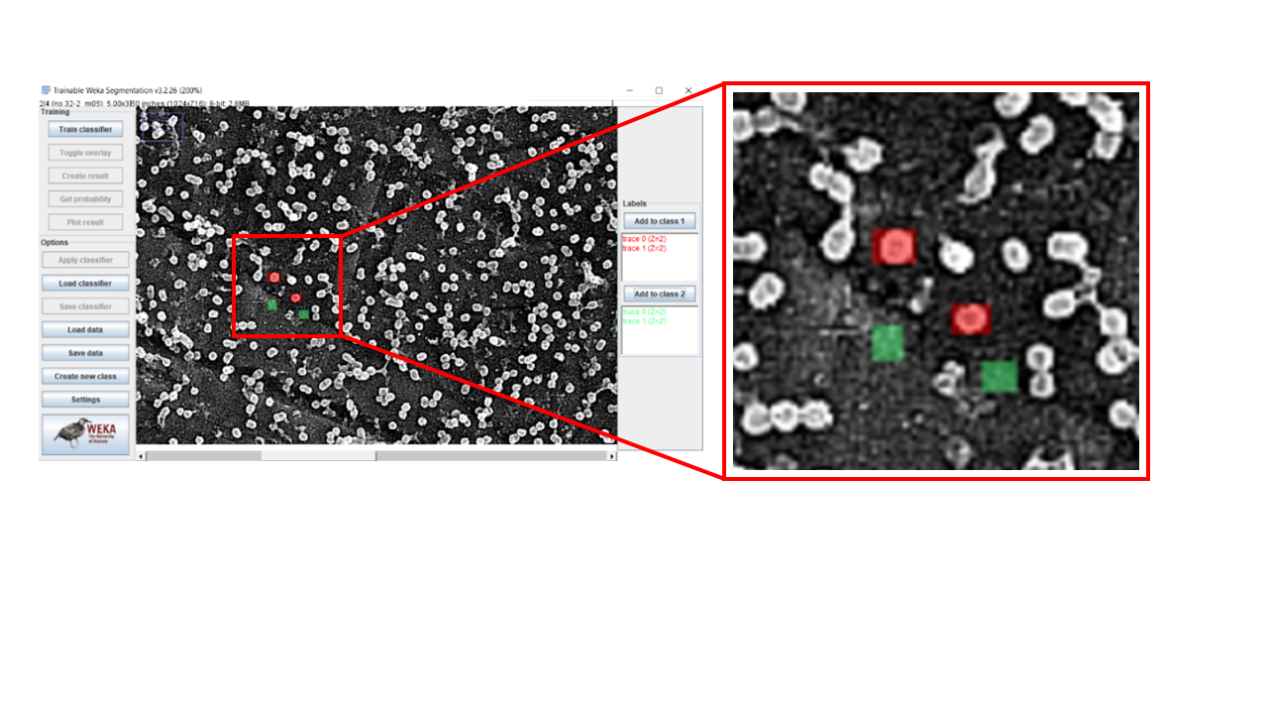


Figure S4 Machine learning algorithm training.

The trained classifier can be saved for further use. The result distribution would be overlapped with the image once finished.


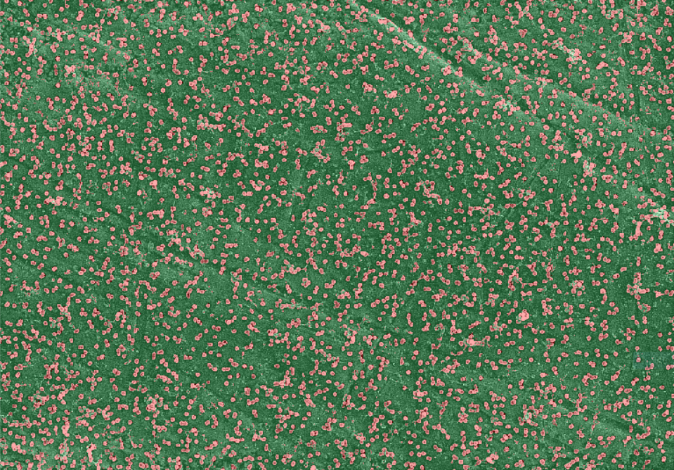


Figure S5 Training result overlapped with SEM image.

Press “Create result”, a classified image with distribution will be created.

As Figure S6 shows, the image will then be converted to a black and white 8-bit image (Image 🡪 Adjust 🡪 Threshold 🡪 B&W 🡪 Apply). The black area indicates the bacteria occupied area and the white area indicates the background.





Figure S6 8-bit B&W classified image.

Finally, choose “Analyze Particles” tool (Analyze 🡪 Analyze Particles) to count the amount and area percentage of the bacteria.


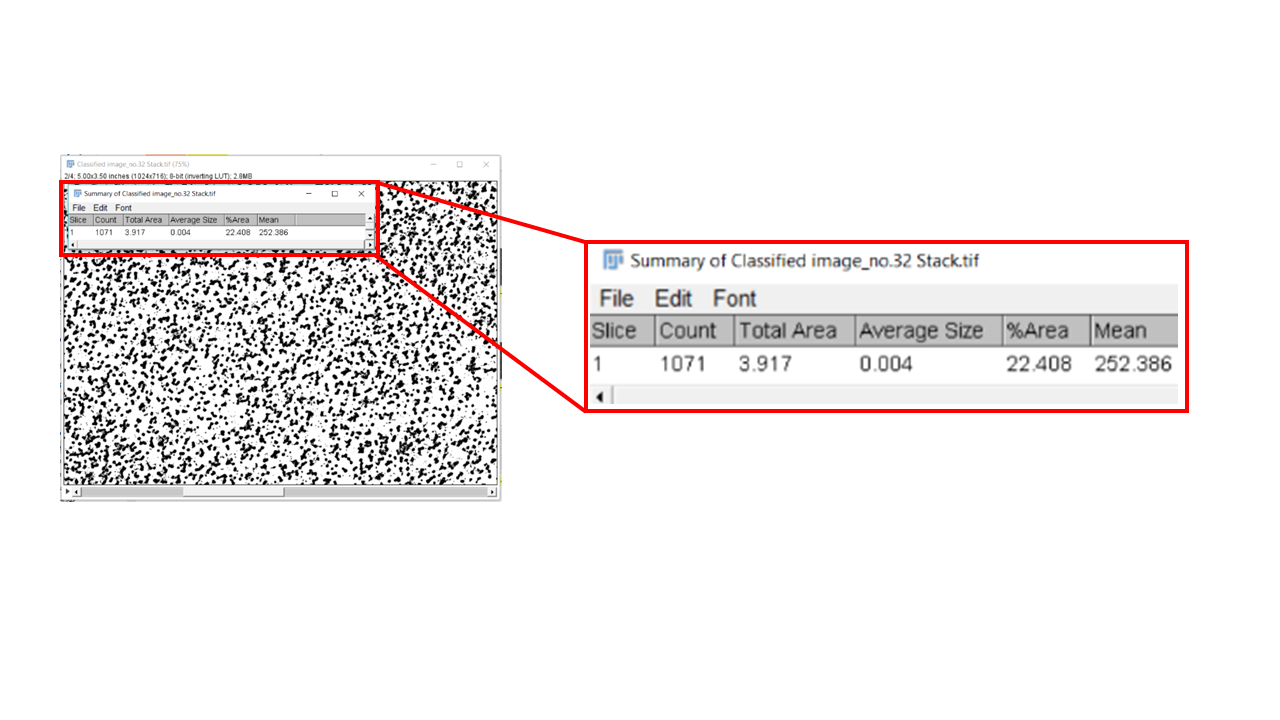


Figure 5 Analyse Results

The unit of “Total Area” and “Average Size” shown in the summary are square pixels, square pixels can be converted into SI base units.
